# Data on consumers’ purchase behavior towards certified rice in Vietnam

**DOI:** 10.1016/j.dib.2021.107010

**Published:** 2021-03-27

**Authors:** Nguyen H.D. My, Matty Demont, Wim Verbeke

**Affiliations:** a Department of Agricultural Economics, Ghent University, Ghent, Belgium; b Faculty of Economics and Development Studies, University of Economics, Hue University, Hue City, Vietnam; c International Rice Research Institute (IRRI), Los Baños, Philippines

**Keywords:** Consumer, Food safety, Certification, Production standards, Rice, Vietnam

## Abstract

The data presented in this article capture purchase behavior of certified rice of 199 urban Vietnamese consumers, surveyed in August 2016. The dataset captures behavioral outcomes in terms of rice purchase as well as factors that affect purchase behavior such as psychological and socio-economic determinants. The data not only contribute to generating a better understanding of the drivers of purchase behavior towards certified rice, but also provide insights into the inclusiveness of consumer access to food safety for staple crops such as rice in Vietnam. Our data and survey instrument may serve as a reference for other developing countries with a similar context and facing similar challenges.

## Specifications Table

**Table:** 

Subject	Economics, Food Science and Technology, Agricultural Sciences
Specific subject area	Agricultural economics, Food consumer behavior, Sustainable food production and consumption
Type of data	Cross-sectional; Excel data file and survey instrument
How data were acquired	Survey using paper-based questionnaire
Data format	Excel data file, raw and processed
Parameters for data collection	The study applied non-probability quota sampling with age, education, and income as quota control characteristics, aiming to match the distribution of the participants in the sample to the distribution in the population.
Description of data collection	Cross-sectional data were collected in August 2016 through a consumer survey (*n* = 199) in Can Tho, a large and centrally-governed city located in the Mekong Delta in the South of Vietnam.
Data source location	City: Can Tho Country: Vietnam
Data accessibility	Data is available in a public repository. Repository name: Mendeley Data Data identification number: http://dx.doi.org/10.17632/38w64m7w7t.1 Direct URL to data: http://dx.doi.org/10.17632/38w64m7w7t.1
Related research article	My, N.H.D., Demont, M., Verbeke, W. (2021). Inclusiveness of consumer access to food safety: Evidence from certified rice in Vietnam. *Global Food Security, 28*, e100491. https://doi.org/10.1016/j.gfs.2021.100491

## Value of the Data

• The data can be helpful for researchers, policy makers, and value chain actors to better understand rice purchase behavior and its determinants, and to enhance inclusiveness of consumer access to food safety for staple crops such as rice in the context of developing countries such as Vietnam.
• The data serve as the initial effort to validate the conceptual framework for investigation of inclusiveness of consumer access towards certified staple foods in the context of developing countries, as presented in My et al. [1].
• The data are useful in discerning the effect of crucial socio-economic factors such as income in conditioning purchase behavior of certified rice, a leading staple in Vietnam and key contributor to food security worldwide.
• The data can assist policy makers in developing a food quality and safety certification system and related communication strategies for staple crops such as rice in the context of an important rice producing and exporting country such as Vietnam.
• The data can be helpful for researchers in designing future research protocols on consumer purchase behavior of staple foods such as rice in general and on inclusiveness of food safety for food crops in specific and in other developing countries where food safety is a major concern. Replication studies in other developing countries and contexts where food safety is a major concern may benefit from benchmarking with the present data.

## 1 Data Description

The dataset captures behavioral outcomes in terms of rice purchase as well as determinants that affect purchase behavior such as psychological and socio-economic determinants. A total number of 199 participants completed the survey using a paper-based questionnaire in August 2016.

Knowledge towards food quality certification was measured by requesting consumers to indicate their answers as “true” (T) or “false” (F) for several statements related to food quality certification in rice. These included: (i) Certified VietGAP rice ensures that the whole rice production process is controlled to ensure the safety for human consumption (T); (ii) Certified HACCP rice indicates that the rice is safe for human consumption (T); (iii) Certified GAP rice does not take into account the welfare of the workers in the supply chain of the product (F); (iv) Certified VietGAP rice indicates that the rice is produced taking into account the negative impacts of farming on the environment (T); (v) Certified rice standards can only be accredited by the government and not by another third party (F). The binary variable “KNO” is assigned the value of “1” for those who answered all statements correctly and “0” otherwise.

Psychological determinants such as benefit beliefs related to different characteristics of certified rice compared to conventional rice were measured based on Grankvist and Biel [2], Steptoe et al. [3] and Van Loo et al. [4]. Items referring to benefit beliefs included: (i) sensory attributes (i.e. aroma, appearance, texture, and taste); (ii) convenience attributes (i.e. ease of cooking, cooking time, and availability); (iii) health-related attributes (i.e. vitamins and minerals, fiber, and nutritional content); (iv) value for money (“PRICE”); and (v) residue levels (“LRESI”). Reponses were scored on a 7-point interval scale ranging from “much worse” (1) to “much better” (7). Composite construct scores for sensory benefit beliefs, health benefit beliefs, and convenience benefit beliefs were computed (“SENS” (Cronbach's α = 0.79), “HEALTH” (Cronbach's α = 0.62), “CONVE” (Cronbach's α = 0.87)). The binary variable “LRESI” is coded as “1” for those who believe that certified rice has much less residues compared to conventional rice and “0” otherwise.

Adapted from Chryssochoidis [5], participants were asked the extent to which they agree with the following statements related to their perceived self-competence (“COMPT”) in identifying certified rice: (i) I know a lot about certified rice products; (ii) I know how to distinguish certified from conventional rice; (iii) Before I purchase rice I know how to look at the differences between products. Answers were recorded on a 7-point Likert (interval) scale ranging from “strongly disagree” (1) to “strongly agree” (7). A composite construct score “COMPT” was computed (Cronbach's α = 0.89).

Corresponding to Janssen and Hamm [6], participants were asked to indicate the extent to which they trust in the food quality certification system for rice. Answers were on a 7-point interval scale ranging from “completely distrust” (1) to “completely trust” (7). The binary variable “TRUST” corresponds to “1” for those who indicated to completely trust the food quality certification system for rice and “0” otherwise.

Similarly to Abdul-Muhmin [7], participants were asked the extent to which they agree with several statements related to their perceived psychological consequences of environmentally friendly behavior (“ENV”). These included: (i) I would feel a sense of satisfaction if I could separate my garbage for recycling; (ii) I would feel a sense of achievement if I can teach my children to respect the environment; (iii) It would mean a lot to me if I could contribute to protect the environment. Answers were recorded on a 7-point Likert (interval) scale. A composite construct score “ENV” was computed (Cronbach's α = 0.71).

Analogously to Seetisarn and Chiaravutthi [8], participants were asked to indicate how frequently they read food labels while making food purchase decisions. Reponses were scored on a 5-point categorical scale ranging from “never” (1) to “always” (5). The binary variable “READ” takes the value of “1” for those who often and always read food labels while making food purchase decisions, and “0” otherwise.

The importance of the food product as a staple within consumers’ overall diets, i.e. consumption frequency of rice was measured by asking participants to indicate whether or not they eat rice on a daily basis (“1” corresponds to daily consumption of rice; “0” otherwise) (“CONS”).

Based on Van Loo et al. [4], data on participants’ purchase behavior of certified rice was obtained by asking: “Out of 10 times that you buy rice, how often do you choose rice with a certified label?”. The answer was recorded on an 11-point scale ranging from “never” (as left anchor) to “always” (10 out of 10 times) (as right anchor). The binary variable “BUY” of certified rice corresponds to 1 for those who purchased certified rice at least once, and 0 for those who never purchased certified rice. This variable served as the binary dependent variable in the specified probit model in My et al. [1].

Socio-economic characteristics of the participants included age (“AGE”), gender (“GENDER”), income (“INC”), education (“EDU”), having children under the age of 15 years (“CHILD”), and household size (“HHSZ”). The master English version of the survey questionnaire and the dataset are available as supplementary materials in the online version of this data article.

## 2 Experimental Design, Materials and Methods

A consumer survey was conducted in August 2016 (*n* = 199) in Can Tho, a large and centrally-governed city located in the Mekong Delta in the South of Vietnam [1]. Consumers were recruited at the entrance of a large supermarket and asked whether they had 10–15 min to participate in a small survey. Data collected include (i) behavioral outcomes in terms of purchase of certified rice; (ii) psychological determinants; and (iii) socio-economic determinants. Participation was voluntary and participants were allowed to withdraw from the survey at any time. All responses were kept confidential. Data were processed anonymously. Further details about survey materials and methods relating to the dataset are described in My et al. [1]. The determinants of consumer buying behavior towards certified rice were assessed in correspondence with the conceptual framework developed by My et al. [1], based on HLPE [9] and Herforth and Ahmed [10]. Analyses included descriptive statistics and probit modeling using SPSS 22.0 (SPSS Inc., Chicago, IL, USA) and NLOGIT 5.0 (Econometric Software, Inc., Plainview, NY). The data are available as an Excel data file.

## Ethics Statement

At the time the survey was conducted, there was no requirement on the need for formal ethics approval by local authorities. However, the survey was conducted in accordance with the key principles and ethical standards stated in the 1964 Declaration of Helsinki. The collected data about rice purchase behavior and its determinants were not perceived to be sensitive and were considered to be part of participants’ day-to-day routines. Before the start of each interview, the participants were informed about the scope and content of the study, that an interviewer/enumerator would ask them a range of survey questions from a questionnaire, that the survey would take approximately 10–15 min to complete. Participants were informed that the survey was meant for research purposes, their participation was voluntary, they did not need to be an expert on the topic to participate, were allowed to withdraw from the survey at any time without needing to explain and without penalty by advising the researcher/enumerator of this decision, and that all personal information would be kept confidential. The participants gave verbal consent to take part in the study and be interviewed. All responses were kept confidential. Data were anonymized and processed anonymously.

## Declaration of Competing Interest

The authors declare that they have no known competing financial interests or personal relationships which have, or could be perceived to have, influenced the work reported in this article.

## Appendix Supplementary materials

Image, application 1

Image, application 2
