# Supplementary material for: Data on consumers’ purchase behavior towards certified rice in Vietnam
Source: Data Brief. 2021 Mar 27;36:107010. doi: 10.1016/j.dib.2021.107010 (PMC8056399; doi:10.1016/j.dib.2021.107010)
Supplement: Supplementary file 2 [file mmc2.pdf]

ANNEX - QUESTIONNAIRE

I. SECTION 1

1. Indicate if the following statements about certified rice are true or false.

Mark one answer per row.

Statement	True	False	Don't know
1. Certified VietGAP rice ensures that the whole rice production process is controlled to ensure the safety for human consumption	O	O	O
2. Certified HACCP rice indicates that the rice is safe for human consumption	O	O	O
3. Certified GAP rice does not take into account the welfare of the workers in the supply chain of the product	O	O	O
4. Certified VietGAP rice indicates that the rice is produced taking into account the negative impacts of farming on the environment	O	O	O
5. Certified rice standards can only be accredited by the government and not by another third party	O	O	O

2. How would you evaluate the following aspects when comparing certified rice (e.g. GAP) with conventional rice. Below are examples of certified rice: for instance GAP certified rice is rice that follows certifications such as VietGAP, GlobalGAP,...

[Instruction to enumerators: Introduce glossary of certifications/concepts to the participants]

"The …<item>… of certified rice (e.g. GAP) is (worse/better) than conventional rice"

Mark one answer per row.

Item	Much worse	Worse	Somewhat worse	Same	Somewhat better	Better	Much better
Smell / Aroma	O	O	O	O	O	O	O
Looks / Appearance	O	O	O	O	O	O	O
Texture	O	O	O	O	O	O	O
Taste	O	O	O	O	O	O	O
Easy to cook	O	O	O	O	O	O	O
Time to cook	O	O	O	O	O	O	O
Availability (place of purchase)	O	O	O	O	O	O	O
Amount of vitamins and minerals	O	O	O	O	O	O	O
Amount of fibre and roughage	O	O	O	O	O	O	O
Nutritional value	O	O	O	O	O	O	O
Amount of residues (e.g. pesticides)	O	O	O	O	O	O	O
Price	O	O	O	O	O	O	O

Time to cook: worse = longer time needed to cook; better = shorter
Availability: worse = more difficult to find; better= less difficult to find
Price: worse = more expensive/not good value for money; better = less expensive/good value for money

3. Out of 10 times that you buy rice, how often do you choose rice with a certified label such as GAP, HACCP, organic, ...

[Instruction to enumerators: Introduce glossary of certifications/concepts to the participants]

0	1	2	3	4	5	6	7	8	9	10
O	O	O	O	O	O	O	O	O	O	O

0= never, 10= always

4. How often do you eat rice?

Never	Less than 1 day per month	1 day per month	1 day per week	2 to 3 days per week	4 to 5 days per week	Daily or almost daily
O	O	O	O	O	O	O

5. Indicate how much you trust the food quality certification system (e.g. GAP) for rice

Completely distrust	Distrust	Somewhat distrust	Neither trust nor distrust	Somewhat trust	Trust	Completely trust
O	O	O	O	O	O	O

6. Indicate to what extent you agree with the following statements. *Mark one answer per row.*

Statements	Strongly disagree	Disagree	Somewhat disagree	Neither disagree nor agree	Somewhat agree	Agree	Strongly agree
I know a lot about certified rice products	O	O	O	O	O	O	O
I know how to distinguish certified from conventional rice	O	O	O	O	O	O	O
Before I purchase rice I know how to look at the differences between products	O	O	O	O	O	O	O

7. Indicate to what extent you agree with the following statement. *Mark one answer per row.*

	Strongly disagree	Disagree	Somewhat disagree	Neither disagree nor agree	Somewhat agree	Agree	Strongly agree
I would feel a sense of satisfaction if I could separate my garbage for recycling	O	O	O	O	O	O	O
I would feel a sense of achievement if I can teach my children to respect the environment	O	O	O	O	O	O	O
It would mean a lot to me if I could contribute to protect the environment	O	O	O	O	O	O	O

8. How often do you read the food label before making a purchase decision?

Never	Rarely	Sometimes	Often	Always
O	O	O	O	O

II. SECTION 2

1. Gender:
   - O Male
   - O Female

2. Age: ______ years

3. How many children (0-14 years old) are living in your household?

|__|__| children younger than 15

4. What is the highest level of education that you have completed?

- O Elementary school and lower
- O Secondary school
- O High school
- O Higher education (not university)
- O University and upper

5. How would you estimate the financial situation of your family?

- O Difficult
- O Average
- O Better than average
- O Well-off

6. What is the total number of members currently living with you in your household?

.... person(s)

Thank you for completing the questionnaire.

Glossary of certifications/ concepts

VietGAP (Vietnamese Good Agricultural Practices) is a national good agricultural practices standard for food issued by the Ministry of Agriculture and Rural Development. VietGAP consists of different criteria with respect to different agricultural products including vegetables, rice, fruit, etc. This is a food safety control and inspection program, starting from farm preparation, cultivation through to harvesting, post-harvest storage, taking into account the environment, any chemicals used, crop or plant protection products, packaging, as well as working conditions and the welfare of workers on farms.

GLOBALG.A.P. (GLOBAL Good Agricultural Practices) sets voluntary standards for the certification of production processes for agricultural products around the globe, using the production method that minimizes the negative environmental impacts of farming operations, reducing the use of chemical inputs and ensuring a responsible approach to worker health and safety, as well as animal welfare.

Organic food products' certification certifies that no chemical inputs or ingredients from genetically modified organisms have been used during the production process.

HACCP (Hazard Analysis and Critical Control Points) is a process that identifies where potential contamination can occur (the critical control points) and strictly manages and monitors these points as a way of ensuring that the process is under control and that the safest product possible is being produced.
